# Discriminating Fever Behavior in House Flies

**DOI:** 10.1371/journal.pone.0062269

**Published:** 2013-04-19

**Authors:** Robert D. Anderson, Simon Blanford, Nina E. Jenkins, Matthew B. Thomas

**Affiliations:** 1 Department of Entomology and Center for Infectious Disease Dynamics – Penn State University, University Park, Pennsylvania, United States of America; 2 Department of Biology and Center for Infectious Disease Dynamics – Penn State University, University Park, Pennsylvania, United States of America; California State University Fullerton, United States of America

## Abstract

Fever has generally been shown to benefit infected hosts. However, fever temperatures also carry costs. While endotherms are able to limit fever costs physiologically, the means by which behavioral thermoregulators constrain these costs are less understood. Here we investigated the behavioral fever response of house flies (*Musca domestica* L.) challenged with different doses of the fungal entomopathogen, *Beauveria bassiana*. Infected flies invoked a behavioral fever selecting the hottest temperature early in the day and then moving to cooler temperatures as the day progressed. In addition, flies infected with a higher dose of fungus exhibited more intense fever responses. These variable patterns of fever are consistent with the observation that higher fever temperatures had greater impact on fungal growth. The results demonstrate the capacity of insects to modulate the degree and duration of the fever response depending on the severity of the pathogen challenge and in so doing, balance the costs and benefits of fever.

## Introduction

Fever is a highly conserved innate immune response [1]. In many organisms it has been shown that fever temperatures serve to increase host fitness by decreasing the rate of pathogen replication [2], [3] and/or increasing the efficiency of the immune system [4], [5]. However, even relatively brief periods of hyperthermia can impose significant costs on the host [6], [7], [8]. In the case of terrestrial ectotherms, attaining and maintaining fever temperatures requires that significant effort be devoted to basking behaviors, potentially detracting from time normally allocated to other essential functions [9]. In order to limit these costs, theory predicts that organisms should invest in fever according to the level of immune challenge.

Although fever in ectotherms has been well documented, the majority of studies have focused on exploring either the mechanisms of fever [10], [11], [12] or the net benefits of fever by quantifying fitness correlates such as survival and fecundity in fevering and non-fevering hosts [13]. In contrast, few studies have provided observations detailing how freely thermoregulating ectotherms behaviorally manage fever throughout the course of infection.

We have previously shown that house flies generate a behavioral fever when infected with a lethal strain of the entomopathogenic fungus, *Beauveria bassiana* [6], [14]. In this earlier work, fevering did not clear infection but nominally benefited fly fitness by reducing pathogen virulence, extending fly survival and enabling infected females to lay more eggs over their lifetime. However, fevering flies incurred costs in the form of decreased egg viability [6]. In order to balance these costs and benefits, we hypothesize that infected flies should adjust their fever behavior in accordance with the level of infection. To test this hypothesis, we explored how fungal dose affects the thermoregulatory behavior of individual house flies and complemented this behavioral assay with direct measures of the impact of fever temperatures on fungal growth.

## Materials and Methods

### Infecting and Marking House Flies

*Beauveria bassiana* conidia formulations were prepared by suspending dry, pure conidia powder into a mixture of mineral oils (80% Shell-Sol: 20% Ondina oil) according to [6]. Conidial concentrations for each formulation were counted using an improved Neubauer Hemocytometer and the volume adjusted until the desired concentration was obtained. Conidia were shown to have germination rates of over 90% as assessed by plating on Sabouraud Dextrose Agar (SDA). We used two conidial concentrations, which we refer to has the high (5×10^8^ spores/ml) and low dose (1×10^8^ spores/ml). These formulations were applied to paper substrates (9 cm diameter disks of color photocopy paper) using an artist’s airbrush sprayer at an equivalent rate of 20 ml/m^2^. Substrates were dried overnight at room temperature before flies were exposed.

House flies were maintained under standard insectary conditions at 27°C, under a 12L: 12D photoperiod. Within 12 hours of eclosion, individual female flies (n = 75) were anaesthetized with cold, and assigned unique color codes by marking them with a small dot of non-toxic enamel paint on both wing bases and the scutum, which enabled the identification of individual flies. Once dry, 25 flies were immediately exposed to each fungal treatment by enclosing them in petri dishes with the treated paper substrates for four hours. Control flies were treated identically, although exposed to unsprayed substrates.

### Thermal Gradient Boxes

After infection, flies were immediately placed in thermal gradient boxes (TGBs) as described previously [6]. Each TGB consisted of a 30×30×15 cm wood-framed box, sealed on all sides by fiberglass screening except for a small flap, which was held in place with magnetic tape to allow the changing of food and removal of dead flies. The front of the each TGB was covered with a 30 cm^2^ plexiglass sheet, while the back was covered with a 30 cm^2^ piece of aluminum sheet metal (0.5 mm thick) to serve as the gradient surface. A metal soup can was used to house a 60W light bulb that was adhered to the center of the sheet metal back. A continuous dimmer switch spliced into the power cord allowed finely tuned adjustments to heat output. The day prior to placing house flies in the gradient box, TGB’s were placed in a growth chamber to maintain an ambient temperature of 26°C, and the temperature of each gradient box was adjusted so the center of each box stabilized at 50°±0.2 C. The pattern of heat dispersion was then characterized for the gradients by measuring the temperature of the gradient surface at 0, 45, 90, 135, 180, 225, 270 and 315 degrees from the vertical at 1 cm increments from the center. Using these data, the temperature for a given point on the thermal gradient surface could be estimated using polynomial regression:
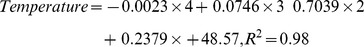



Where x = the distance from the fly to the center of the gradient.

The day after fungal exposure, heat was supplied to each gradient box for six hours daily (8 am–2 pm) starting two hours after the onset and ending four hours before the end of each daily photoperiod. The heat output for each gradient was checked daily within 5–10 minutes of the onset of the heating period to insure that all gradients were providing equivalent amounts of heat. Flies were provided access to food (a 1∶1 ratio of powdered milk and granulated sugar) and water *ad libitum* over the course of the experiment.

### Thermoregulatory Behavior

Each group of flies was placed into a separate thermal gradient box according to treatment. The position of each fly was recorded by taking digital pictures of the gradient surface every ten minutes for the entire six-hour heating period, resulting in a total of thirty-six pictures of each gradient per day. Images were analyzed by importing them into ImageJ version 1.38× [15]. After calibrating the measurement tool to a known distance (1 cm) in each digital picture, the distance from each fly to the center point of the gradient was measured and used to estimate fly temperature using polynomial regression.

### Direct Effects of Fever Temperatures on the Pathogen

Disentangling the direct and indirect effects of fever can be difficult when host and pathogen cannot be examined independently. We have previously shown that the fever provides survival benefits, but also imposes costs on house flies [6]. To determine effects of fever temperatures on the fungus independent of the fly, we measured vegetative growth rates *in vitro* under a range of temperature conditions. For this, a loop of conidial suspension (1×10^7^ conidia/ml prepared in.05% Tween) was placed centrally on agar (SDA) plates and sealed with Parafilm. Four replicate plates were then assigned to one of seven temperature regimes. Control plates were maintained at a constant 26°C. Treatment plates were also maintained at 26°C but exposed additionally to either 37° or 39°C for one, two or three hours daily, representing a range of daily fever intensities observed in flies in the gradient boxes. After 11 days, radial growth of the fungus was measured by taking the average of two perpendicular colony diameters previously marked on each plate prior to inoculation.

### Statistical Analysis

Fly thermoregulatory behavior, including preferred temperatures and proportion of time spent on the gradient surface was analyzed using a repeated measures linear mixed model (LMM) with individual flies serving as replicates (n = 25) in each treatment to allow for missing data due to attrition. Fungal growth data were analyzed using a General Linear Model with time and temperature included as fixed factors. All analyses were performed using SPSS statistical software (version 19) and significance for all tests was set at p<0.05.

## Results

**Figure 1:**
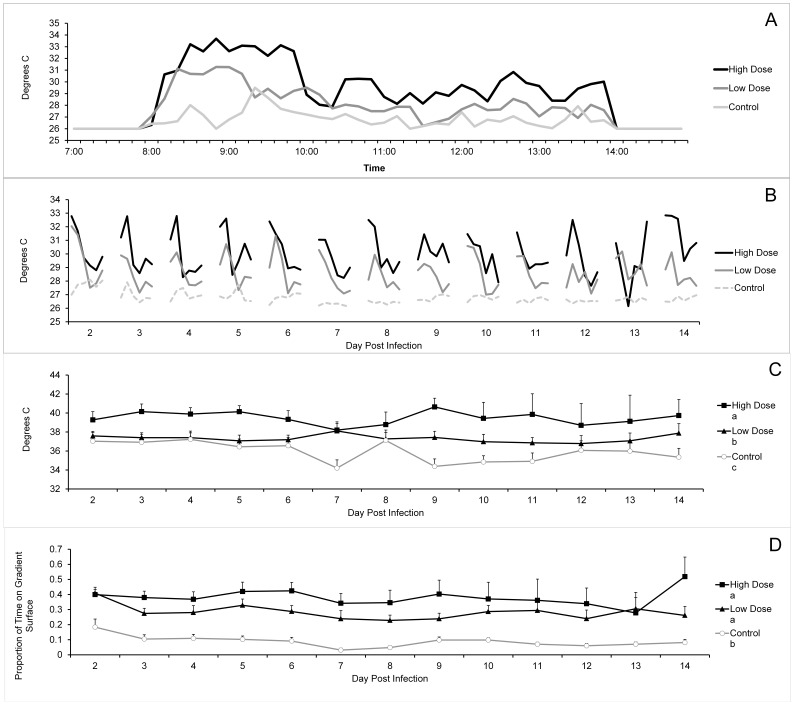
Thermoregulatory behavior of house flies infected with either a high dose (5×10^8^ conidia/ml) or low dose (1×10^8^ conidia/ml) of *Beauveria bassiana*, compared to uninfected flies. A) Represents an example distribution of the hourly mean temperature of flies in each treatment group on the third day of infection, while B) represents the hourly mean temperatures of flies in each treatment group over the course of infection during the time when the gradient was heated (6 hrs a day) over the course of the study. Flies were at ambient temperature (26°C) when they were not sitting on the gradient or when the gradient was turned off. Error bars were not included panel A-B to afford clarity of the fly temperature trends. C) Mean maximum daily preferred gradient temperatures, D) mean daily proportion of time spent on the gradient surface over the course of infection. Bars represent +1 SEM. Groups with different letters in the legends of each graph indicate significant differences (Linear-Mixed Model Repeated Measures ANOVA, sig. *P*<0.05).

The overall pattern of thermoregulation showed flies recruited to a warm part of the gradient at the beginning of the heating period and then gradually redistributed to cooler parts, or left the gradient completely, as the day progressed (Fig. 1a). This general pattern was common across treatments. However, infected flies were found to select warmer areas of the gradient than uninfected controls (F_2, 57.75_ = 21.45, *P*<0.001), with the maximum temperature chosen positively correlated with dose (p’s <0.05, Fig. 1b). In addition, all infected flies remained on the gradient surface for a greater proportion of time than control flies (F_2, 64.64_ = 26.89, *P*<0.001) and flies infected with a higher dose tended to spend more time on the gradient surface than flies infected with the low dose, (*P* = 0.065, Fig. 1c.).

**Figure 2:**
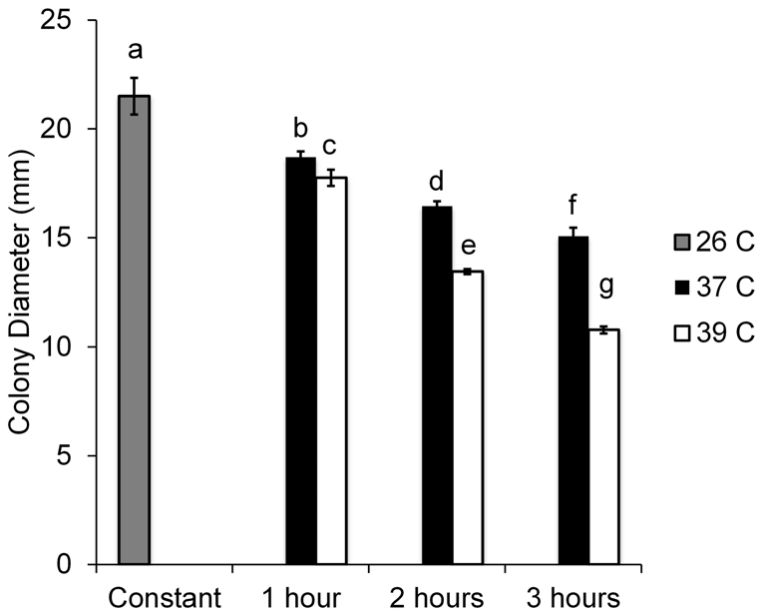
Mean colony diameter of *Beauveria bassiana* grown on agar (SDA) under different temperature regimes over 11 days. Different letters indicate significant differences between groups (Tukey post-hoc test with Bonferroni correction, *P*<0.05). Bars represent ±1 SEM.

Simulated fever treatments impacted fungal growth (Fig. 2), with colony development reduced in all temperature treatments relative to controls (F_6, 27_ = 74.29, *P*<0.0001). There were significant main effects of temperature (F_1,27_ = 66.89, *P*<0.001) and time (F_2,27_ = 57.18, *P*<0.001), as well as a significant interaction term (F_2,27_ = 8.45, *P* = 0.002) indicating that growth was increasingly inhibited as temperature and exposure period increased simultaneously.

## Discussion

Our aim in the current study was to characterize the pattern of behavioral fever in response to different intensities of fungal infection. Fever in houseflies has been shown to provide survival benefits upon fungal infection [6]. Here we demonstrate that part of this benefit likely comes from direct negative effects of high temperatures on fungal growth. The more extreme the ‘fever temperature’, the greater the impact on the fungus, suggesting that higher and more prolonged fevers would be beneficial. However, there are also costs to mounting a fever response [6]. Given both costs and benefits we expect flies to tailor investment in behavioral fever in line with the severity of the immune elicitors causing infection and/or the extent to which temperatures remain conducive to pathogen growth during periods when the houseflies cannot thermoregulate. Consistent with this hypothesis, flies showed temporal variation in fever, selecting the highest temperatures for a relatively short period early in the day and then gradually moving to cooler areas. They also exhibited different intensities of fever, selecting hotter fever temperatures (and possibly investing marginally more time in active thermoregulation) when infected with a higher fungal dose.

Dose-response in fever has been shown previously [10], [11], [16]. However in most of these studies, test organisms were challenged with killed pathogens or pathogen derived immune elicitors to initiate the fever response, thereby eliminating the natural dynamics of infection. In our system, we suggest the degree of fever is likely correlated with the dose of immune elicitors presented by the fungus and so changes with the dynamics of infection. With a higher fungal dose, the magnitude of fever is increased. Similarly, in the early morning when the fungus has likely been growing at its optimal temperature throughout the night cycle, immune elicitors are at high levels and the fever response most intense. As the exogenous immune elicitors are reduced or removed from the hemolymph by the immune response and thermal stress placed on the fungus by fevering flies, the ‘signal’ to fever is attenuated, resulting in the fly gradually moving to cooler areas of the gradient. Overnight, the fungus recovers as the fly cannot fever to suppress fungal growth, and the cycle begins again the next day.

The patterns of fever could also be determined by energetics. Temperatures higher than the normal thermal optimum are energetically costly; it has been demonstrated in both ectotherms and endotherms that increasing body temperatures by only 3–5°C can increase metabolic rate by up to 60% [8], [17]. Therefore, infected flies might only be able to sustain peak fever temperatures for a relatively short time (1–2 hours) at the beginning of the daily heating period (Fig. 1a).

A third possibility is that the thermoregulatory behavior is under some sort of circadian rhythm, resulting in selection of highest temperatures during the morning hours. Elements of innate immune function have been shown to be under rhythmic control in other Diptera, such as *Drosophila* [18] and Anopheline mosquitoes [19]. However, previous work across numerous study systems has yielded little evidence for rhythmic control of thermal preference [20]–[25]. Moreover, circadian control would not account for the differences in intensities of fever in the different fungal treatments.

The current study is one of the first to describe the daily dynamics of fever to a live pathogen and to demonstrate dose-dependence of this immune response in an insect. The molecular pathways responsible for regulating body temperature and fever are highly conserved across taxa [12], [26], [27], implying ancient origins. Endotherms are able to fine tune fever responses depending on the intensity of infection via physiological means [28]. Our results reveal that house flies can similarly tailor patterns of fever via behavioral means, putatively limiting fever costs.
